# Brain Segmentation From Computed Tomography of Healthy Aging and Geriatric Concussion at Variable Spatial Resolutions

**DOI:** 10.3389/fninf.2019.00009

**Published:** 2019-03-18

**Authors:** Andrei Irimia, Alexander S. Maher, Kenneth A. Rostowsky, Nahian F. Chowdhury, Darryl H. Hwang, E. Meng Law

**Affiliations:** ^1^USC Leonard Davis School of Gerontology, Ethel Percy Andrus Gerontology Center, University of Southern California, Los Angeles, CA, United States; ^2^Department of Radiology, Keck School of Medicine of USC, University of Southern California, Los Angeles, CA, United States; ^3^Department of Biomedical Engineering, Viterbi School of Engineering, University of Southern California, Los Angeles, CA, United States; ^4^Faculty of Medicine, Nursing and Health Sciences, Central Clinical School, Monash University, Melbourne, VIC, Australia

**Keywords:** segmentation, tissue classification, computed tomography, multimodal imaging, concussion, geriatrics

## Abstract

When properly implemented and processed, anatomic *T*_1_-weighted magnetic resonance imaging (MRI) can be ideal for the noninvasive quantification of white matter (WM) and gray matter (GM) in the living human brain. Although MRI is more suitable for distinguishing GM from WM than computed tomography (CT), the growing clinical use of the latter technique has renewed interest in head CT segmentation. Such interest is particularly strong in settings where MRI is unavailable, logistically unfeasible or prohibitively expensive. Nevertheless, whereas MRI segmentation is a sophisticated and technically-mature research field, the task of automatically classifying soft brain tissues from CT remains largely unexplored. Furthermore, brain segmentation methods for MRI hold considerable potential for adaptation and application to CT image processing. Here we demonstrate this by combining probabilistic, atlas-based classification with topologically-constrained tissue boundary refinement to delineate WM, GM and cerebrospinal fluid (CSF) from head CT images. The feasibility and utility of this approach are revealed by comparison of MRI-only vs. CT-only segmentations in geriatric concussion victims with both MRI and CT scans. Comparison of the two segmentations yields mean Sørensen-Dice coefficients of 85.5 ± 4.6% (WM), 86.7 ± 5.6% (GM) and 91.3 ± 2.8% (CSF), as well as average Hausdorff distances of 3.76 ± 1.85 mm (WM), 3.43 ± 1.53 mm (GM) and 2.46 ± 1.27 mm (CSF). Bootstrapping results suggest that the segmentation approach is sensitive enough to yield WM, GM and CSF volume estimates within ~5%, ~4%, and ~3% of their MRI-based estimates, respectively. To our knowledge, this is the first 3D segmentation approach for CT to undergo rigorous within-subject comparison with high-resolution MRI. Results suggest that (1) standard-quality CT allows WM/GM/CSF segmentation with reasonable accuracy, and that (2) the task of soft brain tissue classification from CT merits further attention from neuroimaging researchers.

## Introduction

The clinical use of computed tomography (CT) for patient diagnosis and treatment has been increasing steadily throughout the past few decades, particularly in relation to stroke and traumatic brain injury (TBI) (Haydel et al., 2000; Pelc, 2014). In developed countries, the number of CT scanners greatly exceeds that of magnetic resonance imaging (MRI) machines, and CT may be preferable to MRI in emergency radiology settings due to the former modality's convenience, wide availability and speed (Seo et al., 2008). Nevertheless, the task of classifying soft brain tissues based on CT images has long been disregarded because white matter (WM) and gray matter (GM) have relatively poor contrast in CT compared to *T*_1_- or *T*_2_-weighted MRI. The primary reason for this is that soft brain tissues have relatively similar radiodensities, which means that conventional CT images acquired at standard radiation doses typically differentiate GM from WM rather poorly. This frequently makes the CT-based delineation of WM/GM boundaries difficult and inaccurate; if hard thresholds of image intensity are used as primary criteria for delineation, poor GM/WM contrast can lead to substantial error during tissue segmentation. For similar reasons, models involving seed-based region-growing techniques can also lead to misleading results.

Whereas the automatic segmentation of brain MRI volumes is relatively routine compared to CT segmentation (Friston, 2007; Jenkinson et al., 2012; Velasco-Annis et al., 2017), there are very few software solutions for CT-based brain tissue classification. Nevertheless, recent progress in CT scanner technology and the accompanying improvement in CT image quality both suggest that the ability to distinguish soft tissue types using CT is becoming increasingly feasible (Li et al., 2014). As of the date of this study, only a handful of automatic methods for CT brain tissue segmentation exist, none of which have been applied to or validated on neurotrauma patients. Gupta et al. (2010), for example, proposed a heuristic segmentation method which leverages intensity thresholding to distinguish WM from GM and from CSF. The efficacy of this method, however, was only postulated based on manually-contoured, high-confidence fiducial brain regions and in the absence of independent confirmation by other imaging techniques. By contrast, Kemmling et al. (2012) introduced a probabilistic atlas based on previously-segmented MRI volumes which was co-registered to CT images to perform tissue classification, but no validation or quantitative evaluation of this approach was implemented in their study. More recently, Manniesing et al. (2017) proposed a method for CT-based segmentation which requires manual corrections using dedicated software and which also relies on the averaging of CT volumes acquired longitudinally from the same subject after the administration of a contrast agent to improve SNR. The accuracy of these authors' approach is unknown in the scenario where no more than one CT scan is available, as in our case. Furthermore, averaging of longitudinally-acquired CT volumes may produce undesirable results in cases where pathology evolution between time points modifies brain shape and structure, such as in TBI or stroke. Additionally, the method of Manniesing et al. involves the segmentation of GM, WM and CSF from *contrast* CT.

The premise of the present study is that brain segmentation methods for MRI hold considerable potential for adaptation to CT image processing. Specifically, our purpose here is to illustrate how two standard MRI analysis methods—namely (A) probabilistic, atlas-based classification and (B) topologically-constrained tissue boundary refinement—can be combined to delineate WM, GM and cerebrospinal fluid (CSF) from head CT images. In MRI analysis, voxel intensities are often modeled using a mixture of Gaussian random variables and tissue classification can be performed within a Bayesian framework. The probability that each voxel belongs to a certain tissue class is then calculated based on anatomic priors, and class membership is assigned based on this probability. In CT, however, where GM/WM contrast is typically quite poorer than in MRI, this approach can frequently result in spurious, anatomically-implausible class membership assignments for voxels near tissue boundaries. We propose to address this shortcoming by applying a standard approach to the neuroanatomy-constrained correction of tissue boundaries based on the local topological properties of the GM/WM interface. Because this method was previously applied only to MRI, part of our study's novelty involves its application to CT.

The feasibility and utility of the segmentation approach illustrated here are revealed by direct comparison of MRI vs. CT segmentations in a group of concussion victims from whom both standard-quality CT and *T*_1_-weighted MRI were acquired. Here and throughout, “standard-quality CT” refers to CT images acquired at radiation dosages which are typical of routine clinical scans in the United States (~2 mSv). Because radiation dosage is intimately related to CT signal quality and to the signal-to-noise ratio (SNR) of CT images, the utility of contrast-based approaches to brain segmentation is substantially dependent upon radiation dosage. In this context, applying our method to CT scans acquired at a standard radiation dosage is critical for highlighting the broad applicability of the segmentation approach.

To our knowledge, this is the only CT segmentation method to undergo rigorous within-subject comparison with high-fidelity MRI. Furthermore, none of the existing CT methods has been used to segment the brains of older adults or of concussion victims. Both qualitative and quantitative comparison of CT- vs. MRI-based segmentations of WM, GM, and CSF indicate noteworthy agreement between the two, as well as superior segmentation quality compared to the very few other methods currently available. On the other hand, our findings also suggest that—although the reliable CT-based calculation of WM/GM/CSF volumetrics is feasible at standard radiation dosages—the accuracy of CT-derived metrics is unlikely to ever surpass that of MRI-derived ones as the “gold standard” in the field. Scientists who wish to use CT-based volumetrics to make scientific inferences should be mindful that CT-based volumetrics are likely associated with greater error than MRI-based measures. Awareness of this is necessary to prevent future CT-based segmentation studies from conveying an overly optimistic impression regarding the ability of CT segmentations to furnish reliable estimates of brain volumetrics.

## Materials and Methods

### Participants

This study was carried out in accordance with the recommendations of the Institutional Review Board of the University of Southern California with written informed consent from all subjects. All subjects gave written informed consent in accordance with the Declaration of Helsinki. The protocol was approved by the Institutional Review Board at the University of Southern California. Study participants were selected from two volunteer pools, namely (i) concussion victims who had participated in an unrelated study, and (ii) individuals who had been scanned using MRI/CT for clinical treatment unrelated to this study. To be included, patients had to (A) have had both MRI and CT volumes acquired and available, (B) be 50 years of age or older at the time of their initial brain scan, and (C) exhibit no gross head pathology detectable using CT or MRI at scan time. The exclusion criteria were (A) unavailability of MRI and/or CT data; (B) patient age under 50 years; (C) the existence of substantial, gross head pathology at scan time, as detected via CT and/or MRI and (D) poor CT/MRI data quality (e.g., visually detectable artifacts of any kind). The concussed group included 10 participants (5 males; age: mean μ = 65 years; standard deviation σ = 7 years; range: 54–75 years). The non-concussed group included 25 participants (12 males; age: μ = 61 years; σ = 9 years; range: 52–83 years). Volunteers under the age of 50 were excluded because of our desire to test our method on brains with variable degrees of atrophy. The most important difference between the two groups pertains to the spatial resolution of CT/MRI data. Specifically, in the concussed volunteer group, CT slice thickness was 1.25 mm and MRI slice thickness was 1 mm; in the non-concussed volunteer group, slice thickness was 3.75 mm for CT and 5 mm for MRI. This selection of data was intentional, as the difference in spatial resolution allowed us to explore segmentation reliability as a function of slice thickness and to illustrate the necessity of evaluating CT segmentation approaches like ours using MRI of research-grade resolution.

### Data Acquisition

All data were deidentified and delinked prior to analysis. CT volumes were acquired using a 16-slice General Electric scanner. In the concussed volunteer group, images were acquired clockwise, in helical mode, with a standard convolution kernel and the following parameters: matrix size = 512 × 512; voxel size = 1.5 mm × 1.5 mm × 1.25 mm; kilovoltage peak (kVp) = 120 kV; data collection diameter = 500 mm; exposure time = 600 ms; X-ray tube current = 100 mA; exposure = 100 mA·s; focal spot = 1.2 mm. MRI volumes were acquired at 3 T using a Prisma MAGNETOM Trio TIM scanner (Siemens Corp., Erlangen, Germany). Images were acquired using a magnetization-prepared rapid acquisition gradient echo (MP-RAGE) sequence with the following parameters: repetition time (*T*_*R*_) = 1,950 ms; echo time (*T*_*E*_) = 3 ms; inversion time (*T*_*I*_) = 900 ms; flip angle (FA) = 9 degrees; percentage sampling = 100; pixel bandwidth (BW) = 240 Hz/pixel; matrix size = 256 × 256; voxel size = 1 mm × 1 mm × 1 mm. In the non-concussed volunteer group, CT volumes were acquired clockwise, in helical mode, with a standard convolution kernel and the following parameters: matrix size = 512 × 512; voxel size = 1.5 mm × 1.5 mm × 3.75 mm; kVp = 120 kV; data collection diameter = 250 mm; exposure time = 750 ms; X-ray tube current = 220 mA; exposure = 130 mA·s; focal spot = 1.2 mm. MRIs were acquired at 3 T using a Signa HDxt scanner (General Electric Corp., Boston, USA). Images were acquired using a fast spin-echo (FSE) sequence with the following parameters: *T*_*R*_ = 567 ms; *T*_*E*_ = 18 ms; FA = 90 degrees; percentage sampling = 100; pixel BW = 81 Hz/pixel; matrix size = 512 × 512; voxel size = 0.5 mm × 0.5 mm × 5 mm.

### MRI Segmentation

MRI volumes were segmented using the widely-utilized FreeSurfer 6.0 software as detailed elsewhere (Dale et al., 1999; Fischl et al., 1999), with default execution parameters. Very briefly, this process includes (1) the removal of non-brain tissue using a hybrid watershed/surface deformation procedure, (2) automated Talairach space transformation, (3) volume intensity normalization, (4) segmentation of cortical and subcortical GM, (5) tessellation of the GM/WM boundary, and (6) automated surface topology correction. The reader is referred to references (Dale et al., 1999; Fischl et al., 1999) for comprehensive details on each of these steps involved in the MRI segmentation procedure.

### CT Segmentation

As previously stated, an important goal of this study is to illustrate how MRI-tailored approaches can be combined and adapted for CT. Because of this, our segmentation strategy is inspired by MRI-tailored approaches to template-based tissue classification, including pioneering approaches by Ashburner and Friston (1997, 2000, 2005, 2007) and by Dale et al. (1999) and Fischl et al. (1999). The starting point for our implementation was the probabilistic classification method of Ashburner and Friston (2005), as available in SPM 12.0; this was adapted, modified and augmented in MATLAB to incorporate topology-constrained segmentation (Dale et al., 1999; Fischl et al., 1999). An overview of the entire tissue classification procedure is provided in this section, and details specific to each step are described in subsequent sections. Briefly, to perform tissue classification, voxel intensity values are used to assign their probabilities of belonging to one of several tissue classes by estimating the parameters of the intensity distributions of each class. This is accomplished by first defining an objective function derived from a mixture of Gaussian random variable models, and by then minimizing the value of this function using a parameter optimization process. A set of *a priori* tissue probability maps specified in a standard space (atlas) are used to assist the classification. The objective function can assist this process by weighing the probability maps of the standard space according to Bayesian inference principles and then deforming them so that they match the volumes being segmented. Specifically, the template is warped to each subject's brain volume (Collins et al., 1995), after which the latter can be segmented and the ensuing spatial classifications can be smoothed (Evans et al., 1994). When combined with *a priori* information specified by the template, Bayesian inference can be used to calculate posterior probabilities, based on each subject's voxel intensity values. The interface between the resulting GM and WM volumes is smoothed according to principles inspired from nonlinear filter theory, subject to topological constraints dictated by the structural neuroanatomy of the human brain (Dale et al., 1999; Fischl et al., 1999).

### Gaussian Mixture Model

The distribution of image intensities in a neuroimaging volume is modeled here by a mixture of *K* clusters, each consisting of Gaussian random variables (Bishop, 1995). Each Gaussian variable is parameterized by its mean *μ*_*k*_, variance $\sigma_{k}^{2}$ and mixing coefficient *γ*_*k*_, subject to the constraint that the sum of all mixing coefficients must be equal to 1. Fitting this Gaussian mixture model to the image intensity data vector **y** of length *I* involves maximizing the probability of observing the data given the model parameterization. The probability that a voxel has intensity *y*_*i*_ given that it belongs to the *k*-th Gaussian random variable (i.e., given that *c*_*i*_ = *k*) parameterized by *μ*_*k*_ and $\sigma_{k}^{2}$ is

(1) $$\begin{array}{r} P\left(y_{i}|c_{i}=k,\mu_{k},\sigma_{k}^{2}\right)=\frac{1}{{\left(2\pi \sigma_{k}^{2}\right)}^{1/2}}\exp\left[-\frac{{\left(y_{i}-\mu_{k}\right)}^{2}}{2\sigma_{k}^{2}}\right]. \end{array}$$

Because the probability that *y*_*i*_ belongs to the *k*-th Gaussian random variable given the proportion *γ*_*k*_ of voxels which belong to that random variable is *P*(*c*_*i*_ = *k*|*γ*_*k*_), Bayes' rule indicates that

(2) $$\begin{array}{r} P\left(y_{i},c_{i}=k|\mu_{k},\sigma_{k}^{2},\gamma_{k}\right)=P\left(y_{i}|c_{i}=k,\mu_{k},\sigma_{k}^{2}\right)P\left(c_{i}=k|\gamma_{k}\right), \end{array}$$

and the total probability of observing *y*_*i*_ becomes

(3) $$P\left(y_{i}|\mu ,\sigma ,\gamma \;\right)=\sum_{k=1}^{K}P\left(y_{i},c_{i}=k|\mu_{k},\sigma_{k}^{2},\gamma_{k}\right),$$

whilst the probability

(4) $$P\left(\text{y}|\mu ,\sigma ,\gamma \right)=\prod_{i=1}^{I}P\left(y_{i}|\mu ,\sigma ,\gamma \right)$$

that all *I* intensities in **y** are observed given ***μ***, ***σ***, and ***γ*** can be maximized by varying the latter parameters in the cost function

(5) E=−logP(y|μ,σ,γ).

### Spatial Priors, Deformation, and Regularization

A probabilistic atlas is used to specify the prior probability that each voxel belongs to any tissue class in the Gaussian mixture model. This is done without assuming that any intensity distribution for each class is Gaussian, such that the prior probability of voxel *i* being drawn from the *k*-th Gaussian distribution is

(6) $$\begin{array}{r} P\left(c_{i}=k|\gamma \right)=\;\frac{\gamma_{k}P_{ik}}{\sum_{j=1}^{K}\gamma_{j}P_{ij}}, \end{array}$$

where *P*_*ik*_ is the tissue probability for class *k* at voxel *i*. For voxels located at the boundary between tissues (e.g., the GM/WM boundary), this model accommodates the difficulty of ascertaining the class to which voxel *i* belongs. The atlas used here is a modified version of the MNI_152_ atlas (Grabner et al., 2006), which is based on an average of *T*_2_-weighted MRI volumes acquired from 152 healthy control subjects. The original atlas has a resolution of 1 mm × 1 mm × 1 mm and its image intensities range from 0 to 90 in increments of 1.3 × 10^−3^. For the present study, the atlas in question was modified to reflect the intensity profile of CT brain scans, where CSF is hypointense.

Let ***α*** be a vector of diffeomorphic deformation parameters which allow the co-registration of the spatial template and a subject volume. Here, spatial priors are deformed according to ***α***, to allow co-registration according to

(7) $$P\left(c_{i}=k|\gamma ,\;\alpha \right)=\frac{\gamma_{k}P_{ik}(\alpha )}{\sum_{j=1}^{K}\gamma_{j}P_{ij}(\alpha )}.$$

With this adjustment, one obtains

(8) $$E=-\sum_{i=1}^{I}\log P\left(\text{y}|\mu ,\sigma ,\gamma ,\alpha \right)$$

or, more explicitly,

(9) $$\begin{array}{r} E=-\overset{I}{\underset{i=1}{\sum }}\log\overset{K}{\underset{k=1}{\sum }}P\left(c_{i}=k|\gamma ,\;\alpha \right)P\left(y_{i}|c_{i}=k,\mu_{k},\sigma_{k}^{2}\right). \end{array}$$

The parameterization of the deformation is implemented using a linear combination of sinusoidal transform bases (Christensen et al., 1994) subject to spatial regularization by maximizing *P*(**y**, ***α***|***μ***, ***σ***, ***γ***). Only the lowest frequencies of a discrete sine transform were used, resulting in 392 (7 × 3 × 8) parameters to describe deformations along each spatial dimension. Three additional parameters were used to model linear scaling and one parameter was used to model linear image intensity inhomogeneities (Ashburner and Friston, 1999). The probability densities of the spatial parameters ***α*** are modeled by multivariate Gaussian random variables with mean **0** and covariance matrices **C**_***α***_. The spatial regularization involving these covariance matrices and deformations prevents undesirable interactions between parameter estimates (Evans et al., 1994). Initially, parameter value estimates are assigned randomly, and nonlinear deformation coefficients are set to zero. Model parameters are then optimized using an expectation maximization (EM) algorithm (Bishop, 1995), where the Gaussian mixture and deformations are re-calculated by iteratively updating exactly one while the others are held constant. Deformations are optimized using a Gauss-Newton scheme (Wedderburn, 1974).

### Topology-Constrained Refinement

After probabilistic assignment of voxels to one of three classes (WM, GM or CSF), the segmentation is refined iteratively using *a priori* information concerning the local properties of the cortex (Dale et al., 1999). Specifically, because the surface defined by the WM/GM interface is smooth and its curvature is both defined and finite everywhere on it, the local topology of the brain can be used to correct the probabilistic tissue classification. This process is analogous to the application of a nonlinear, anisotropic filter whose nonlinearity is high near the WM/GM boundary. As the distance from some given voxel to the WM/GM boundary increases, the filter becomes more linear; because the true boundary is topologically smooth, the filter shape must be planar at this interface. In our approach, the segmentation is corrected in two steps. First, we identify the plane crossing the boundary which is intersected by voxels whose intensity variance is minimal. Once this is done, the voxels within this plane are examined to determine whether (A) a substantial proportion of them have ambiguous classifications based on their intensity or whether (B) they are surrounded by voxels whose class memberships vary greatly. If changing the class assignment of these voxels decreases the in-plane intensity variance, the voxels in question are re-assigned to their more appropriate class (Dale et al., 1999).

### Qualitative Segmentation Comparison

CT segmentations were compared to MRI-based segmentations within each participant. Prior to this comparison, the skull-stripped MRI and CT volumes were co-registered using a 12-parameter, affine registration. MRI- and CT-based segmentations were compared by plotting both and inspecting the ability of the CT segmentation to reproduce cortical folding patterns and to identify landmarks of interest, including the thalamus, ventricular system, and various gyri. To visually inspect the effect of slice thickness upon segmentation, the CT volume of a representative concussion victim was first down-sampled using trilinear interpolation to change the voxel size from 1 mm × 1 mm × 1.25 mm to 1 mm × 1 mm × 3.75 mm. The lower-resolution volume was then segmented, and the results were compared.

### Quantitative Segmentation Comparison

In addition to comparing the CT- and MRI-based GM, WM and CSF classifications qualitatively, four measures were calculated: (1) the Sørensen-Dice coefficient (which conveys the extent of overlap between CT and MRI tissue label maps), (2) the Hausdorff distance (which measures, in this case, how far the CT- and MR-based boundaries are between two tissues), (3) the intraclass correlation coefficient (a measure of how reproducible measurements are when made using distinct techniques) and (4) the stretching distance (a measure of average spatial prior deformations).

For two tissue classes *X* and *Y*, the Sørensen-Dice coefficient *C*_*SD*_ is defined as

(10) $$\begin{array}{r} C_{SD}=2\frac{\left|X\;\cap \;Y\right|}{\left|X|\;+\;|Y\right|}. \end{array}$$

If there is perfect overlap between the two tissue classes, *C*_*SD*_ is equal to 1; no overlap results in *C*_*SD*_ being equal to 0. The original Hausdorff distance *d*_*H*_ is defined as

(11) $$d_{H}(X,Y)=\max\left\{{\text{sup}}_{x\epsilon X}{\text{inf}}_{y\epsilon Y}\;d\left(x,y\right),{\text{sup}}_{y\epsilon Y}{\text{inf}}_{x\epsilon X}\;d\left(x,y\right)\right\}$$

where *X* and *Y* are non-empty sets of a metric space (*M, d*), sup is the supremum and inf is the infimum. This measure involves the distance between points located along the edges of two surfaces and conveys how well the two surfaces overlap. In the present study, *X* and *Y* are MRI- and CT-derived segmentation volume surfaces, respectively, and *d* is a Euclidian distance. Here, the modified Hausdorff distance is used, as defined formally elsewhere (Dubuisson and Jain, 1994).

The intraclass correlation coefficient *r*_*IC*_ is a measure of within-subject measurement variability relative to between-subject variability (Iscan et al., 2015). In the present case, these measurements are volumes of the GM, WM, or CSF computed from either MRI or CT, and their *r*_*IC*_ value can be used to quantify the reliability of the CT segmentation. As reported elsewhere, the calculation of *r*_*IC*_ is predicated upon experimental design and statistical model assumptions (Shrout and Fleiss, 1979). In cases like ours, the one-way random effect model is appropriate (McGraw and Wong, 1996), such that *r*_*IC*_ ≃ (*MS*_*b*_ − *MS*_*w*_) /*MS*_*b*_, where *MS*_*b*_ and *MS*_*w*_ are between- and within-group mean sums of squared measurements, respectively. These quantities were computed like in an analysis of variance (Shou et al., 2013). Bootstrapping was used to calculate the average amount by which CT volume estimates can be expected to deviate from their MRI-derived values.

To assess the relationship between segmentation quality and the amount of deformation applied to the spatial priors, one can calculate the mean absolute stretching distance *d*_*S*_ between two volumes (Ewert et al., 2019). Intuitively, this distance can be conceptualized as the average amount by which volume elements within a moving volume must move to match the shape of a target volume. The mapping between volume elements in the two volumes (template and subject) is specified by the deformation field of the transformation. In other words, *d*_*S*_ is the average amount by which voxels in the atlas must move to optimize the atlas-subject deformation. The larger the deformation, the greater *d*_*S*_.

To determine whether outliers as well as any bias existed in favor of any of the segmentation classes, the MRI- and CT-derived volumes of WM, GM, and CSF were plotted against each other. The relationships between *d*_*H*_ and *C*_*SD*_, and between *d*_*H*_ and *d*_*S*_ were explored visually in a similar way, i.e., by plotting one against the other. In this study, all GM and WM measures were calculated based on all neuroanatomical structures in the cranial cavity. By contrast, only ventricular CSF volumes and Sørensen-Dice coefficients were compared because *T*_1_-weighted MRI is insufficiently suited—compared to *T*_2_-weighted MRI—for quantifying water content in the CSF layer around the cerebrum, as well as in locations surrounding the cerebellum, brainstem, etc. However, *T*_2_-weighted MRI scans were unavailable to us; to alleviate this drawback, only ventricular CSF measures were compared across modalities.

In implementations like ours, there is a risk that segmentation results could be dominated by the nonlinear deformation of the template to each individual case. In other words, the radiodensities of distinct tissue classes (e.g., GM, WM) may have relatively little influence upon the segmentation. To test this hypothesis, the following analysis was implemented for each CT volume: (A) The mean μ and standard deviation σ were calculated across all brain CT voxels. (B). All brain voxels were assigned radiodensity values sampled at random from a Gaussian distribution with parameters μ and σ. This operation effectively removed the contrast between GM and WM. (C) The modified brain CT volume was segmented. (D) *C*_*SD*_ and *d*_*H*_ values were computed based on the segmentation of the modified brain CT volume and then compared to the values of these metrics as obtained by segmenting the original CT volume. We argue that, if tissue class radiodensities had no effect upon segmentations, there would be no statistically-significant difference between *C*_*SD*_ values calculated based on original CT volumes vs. based on modified CT volumes.

## Results

### Qualitative Assessment

**Figure 1:**
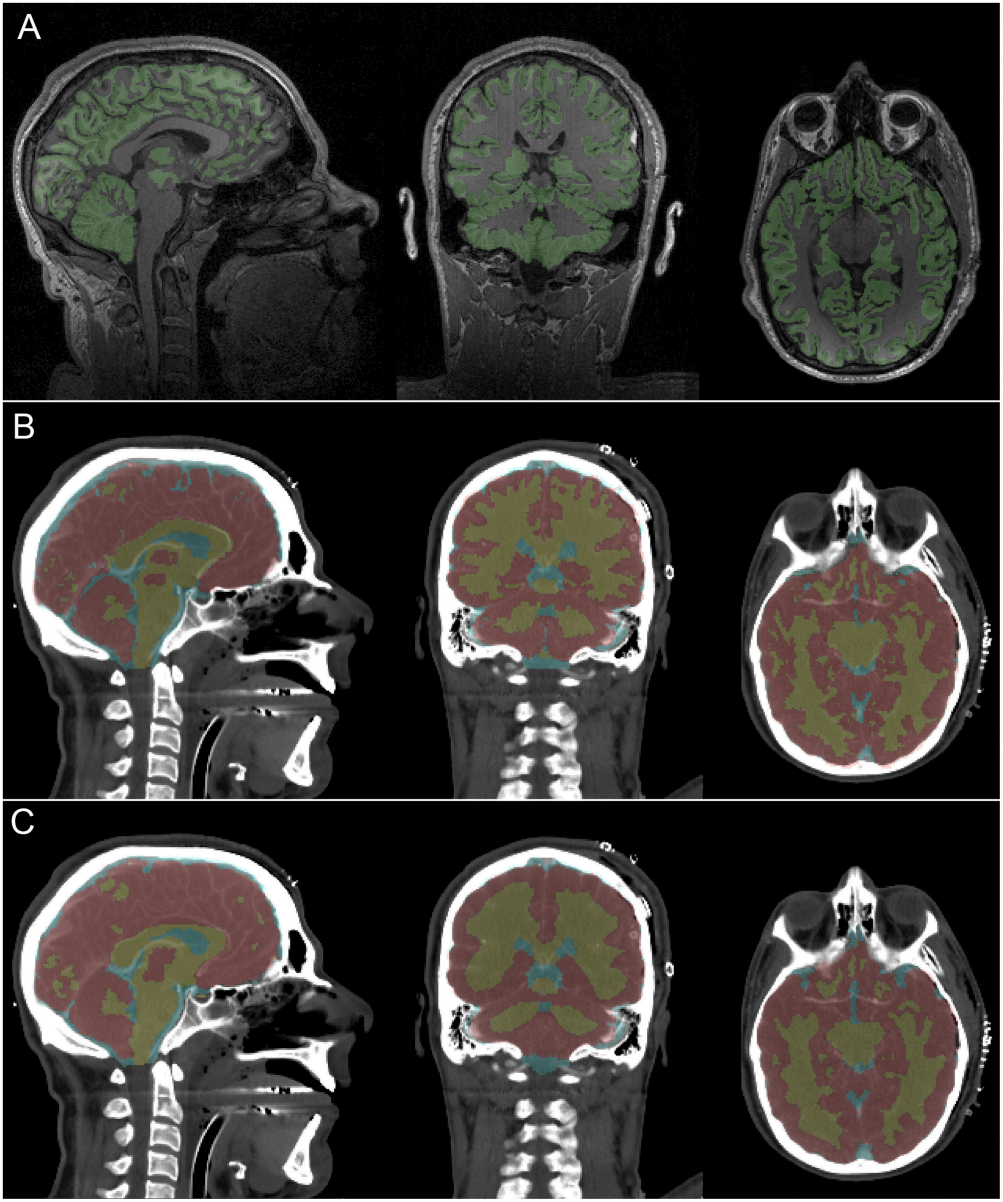
MRI and CT segmentations and their corresponding imaging slices for a representative subject. Colored voxel label maps are translucent to ease inspection of the underlying neuroanatomy. **(A)** *T*_1_-weighted MRI slices show GM (green). The WM is left uncolored to facilitate identifying occasional differences between the true GM/WM boundary and the FreeSurfer-identified boundary. **(B)** CT slices display labeled GM (red), WM (yellow) and CSF (light blue) based on segmentation at the original CT volume resolution (1 × 1 × 1.25 mm). **(C)** Like **(B)**, based on segmentation at a down-sampled CT volume resolution (1 × 1 × 3.75 mm).

The conclusions of our qualitative assessment are reflected by the results conveyed in Figure 1, where both MRI and CT segmentations are displayed for a representative subject. When performing this comparison, the MRI-based segmentation is treated as the gold standard. Overall, the agreement between MRI- and CT-derived classifications is quite reasonable, with our method being able to capture the most prominent features of cerebral neuroanatomy appropriately. In what follows we discuss specific findings, as reflected by the sagittal, coronal and axial views of the brain, respectively.

The sagittal slice of the brain displayed in Figure 1 is approximately co-planar with the longitudinal fissure. This depiction indicates visually-acceptable agreement between the segmentations, with good coverage of cerebral GM, callosal WM and of the brainstem. Ventricular CSF classifications also appear to be satisfactory. There is even agreement between segmentations pertaining to cerebral areas where only little GM is visible in the selected slice, such as the medial parietal lobe and occipital lobes. The most notable difference in the sagittal view pertains to the frontal lobe, where the CT algorithm appears to have classified more tissue along the longitudinal fissure as GM than the MRI method. This, however, is to be expected due to the relatively low SNR of CT compared to MRI as well as to the excellent ability of FreeSurfer software to delineate the natural boundary between hemispheres.

The coronal slice displays a view of the parietal lobe, with a substantial portion of the cerebellum and lateral ventricles being visible as well. This view is particularly useful because it conveys the substantial similarities in gyrification patterns between the two segmentations. Visual assessment confirms that local structural variations are captured relatively well in the CT segmentation. Though the basal ganglia are poorly delineated by CT, our segmentation appears to be able to capture them well. The axial slice is at the level of the inferior temporal lobe, with some frontal lobe structures—such as the orbital gyri/sulci—being visible as well. As in the coronal slice, the overall local shape of the GM/WM boundary is reflected well in the CT segmentation.

**Figure 2:**
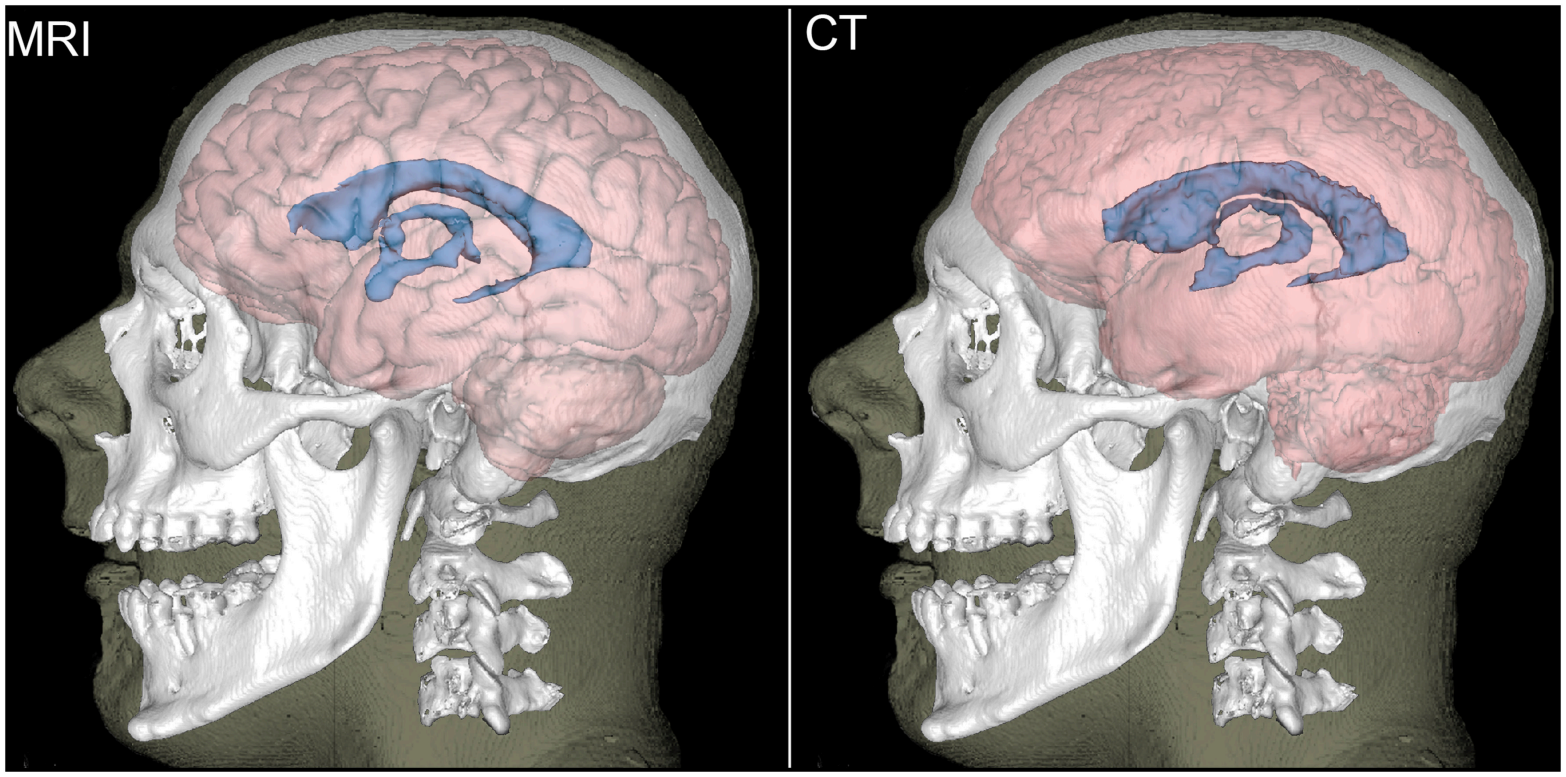
Reconstructions of the brain (light red), ventricular CSF (blue), bones (white), and skin (translucent) for a representative participant. The brain and ventricular CSF are based on MRI **(left)** and on CT **(right)**. Bones and the skin surface were reconstructed from CT.

Figure 2 displays MRI- and CT-based three-dimensional reconstructions of the ventricular CSF, brain, bones and skin for the volunteer in Figure 1. The second row displays segmentation results based on the original-resolution volume (1 mm × 1 mm × 1.25 mm). Although the MRI-based segmentation is superior in its ability to resolve the gyrification of the cortex, the CT segmentation does reproduce the overall shape of the brain and ventricular system. The reconstruction of the lateral ventricles, third ventricle and inter-thalamic adhesion appears to be within reasonable limits for the purposes of neuroanatomic reference and delineation. Results in the third row are based on the same volume after down-sampling to the resolution of the volumes acquired from non-concussed volunteers (1 mm × 1 mm × 3.75 mm). Here, the method is seen to over-estimate GM volume and to lose some ability to capture cortical folding details; overall, there is some perceived loss of tissue classification fidelity compared to MRI.

### Quantitative Assessment of Concussion Group

Across all concussion cases considered, the mean and standard deviation of the Sørensen-Dice coefficient were found to be 86.7 ± 5.6% for WM, 86.0 ± 2.0% for GM, and 92.2 ± 0.7% for ventricular CSF. The means and standard deviations of the coefficient are more similar for WM and GM, presumably because these tissues' similar radiodensities translates into similar abilities to classify them. On the other hand, ventricular CSF has a somewhat greater coefficient presumably because its lower radiodensity compared to GM/WM makes CSF easier to distinguish from soft brain tissue. The average modified Hausdorff distance was found to be 3.4 ± 1.5 mm (WM), 3.7 ± 1.8 mm (GM), and 2.5 ± 1.3 mm (CSF), which confirms that CSF classification is likely best, followed by WM and then GM. This view is recapitulated by the fact that *d*_*S*_ was found to have means of 3.4 ± 2.3 mm (WM), 3.5 ± 1.9 mm (GM), and 1.8 ± 0.6 mm (CSF).

In the concussion sample, the intraclass correlation coefficient was found to be 0.64 for WM, 0.68 for GM, and 0.74 for CSF. Bootstrapping results suggested, based on this sample, that the segmentation method is sensitive enough, to yield WM, GM, and CSF volume estimates within ~5.4%, ~4.3%, and ~3.2% of their MRI-based estimates, respectively. As percentages of the MRI-derived mean volume, the 2σ confidence intervals (CIs) for these error estimates were [2.9, 7.9]% for GM, [2.2, 6.4]% for WM, and [1.4, 5.0]% for CSF. In other words, for a randomly selected volunteer, there was a ~95% estimated probability that the discrepancy between her/his CT-derived and her/his MRI-derived GM volume was between 2.9% and 7.9% of the latter.

**Figure 3:**
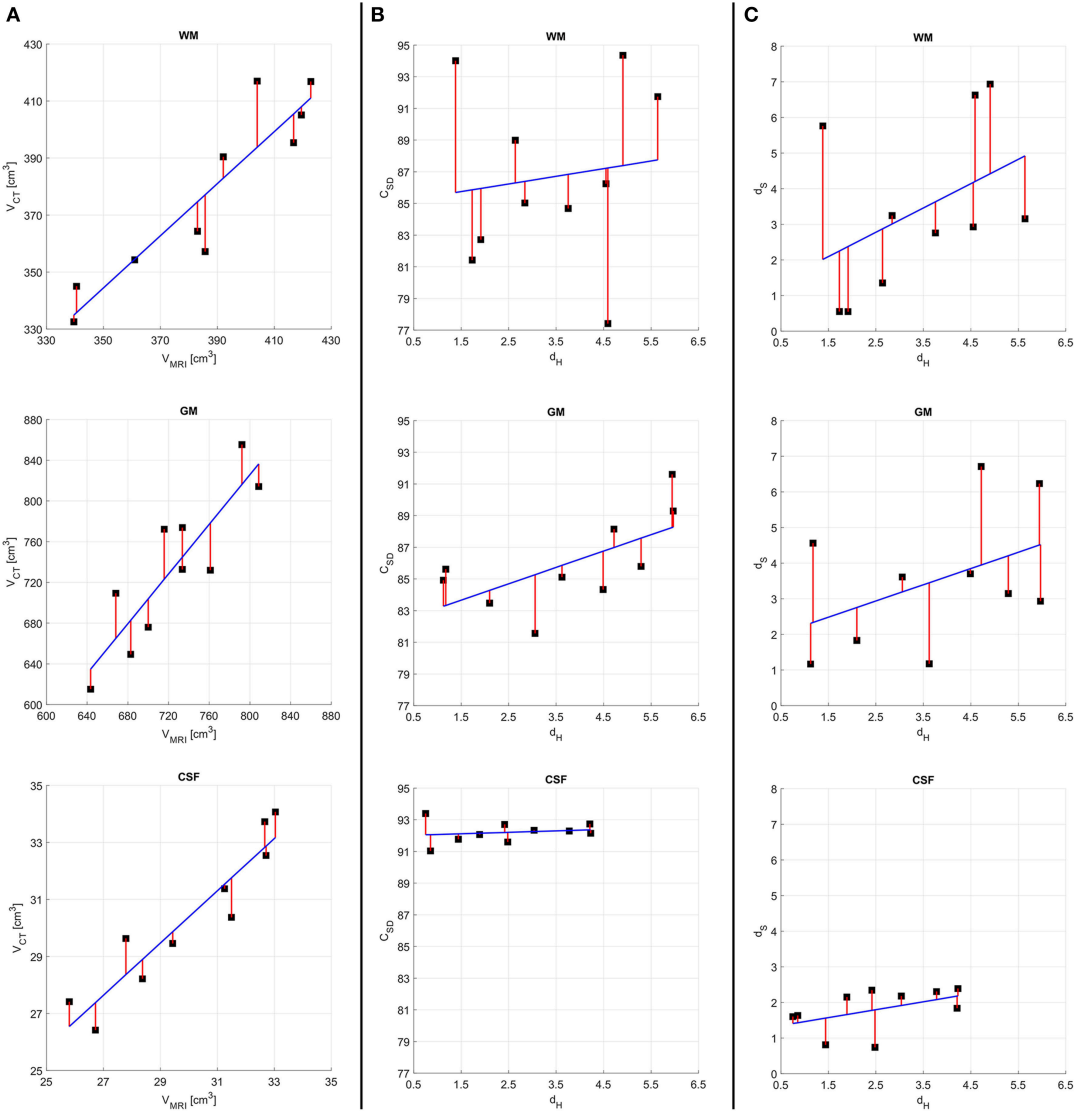
Results of quantitative analysis for concussion victims. For all quantities plotted, the regression line of best fit (blue) and residuals (red) are shown on plots with identical ranges along both *x* and *y*, to facilitate comparison. **(A)** MRI- vs. CT-derived volumes. **(B)** The Hausdorff distance *d*_*H*_ vs. the Sørensen-Dice coefficient *C*_*SD*_. **(C)** The Hausdorff distance *d*_*H*_ vs. the stretching distance *d*_*S*_. Quantities pertaining to WM, GM, and CSF are displayed in the first, second, and third rows, respectively.

The results of the quantitative assessment for concussion victims are summarized in Figure 3. In particular, Figure 3A suggests that, in the case of volume measurements, no outlier or bias in favor of any tissue class are present in our cohort of concussion victims. Figure 3B suggests that, as expected, there is a direct relationship between *d*_*H*_ and *C*_*SD*_. Comparison of the plots for WM, GM and CSF illustrates how both metrics have a smaller range and variance for CSF than for the other two classes. This can be explained by the fact that CSF is easier to segment from both CT and MRI due to the relatively large difference in radiodensity between CSF and either GM or WM. This is confirmed by Figure 3C, where the relationship between *d*_*H*_ and *d*_*S*_ is explored. As expected, these quantities are also directly proportional to each other because they both trend higher as the quality of the segmentation decreases.

When testing the hypothesis that tissue class intensities had no effect upon segmentations, the Sørensen-Dice coefficients computed based on CT volumes with modified radiodensities were found to be 64.2 ± 8.9% for WM and 69.4 ± 7.3% for GM across all concussion cases. The average modified Hausdorff distance was found to be 5.21 ± 1.61 mm (WM) and 4.87 ± 1.95 mm (GM) in this group. These values are significantly different (*p* < 0.001) from those obtained based on the original CT volumes, which suggests that tissue class radiodensities do have a significant effect upon segmentations.

### Quantitative Assessment of Volunteers Without Concussions

Across non-concussed participants (whose MRI volumes had thicker slices), the mean and standard deviation of the Sørensen-Dice coefficient were found to be 63.7 ± 7.2% for WM, 59.4 ± 8.9% for GM, and 73.5 ± 6.6% for ventricular CSF. In this group, the average modified Hausdorff distance was found to be 6.18 ± 2.34 mm (WM), 6.75 ± 2.87 mm (GM), and 4.89 ± 1.86 mm (CSF). Presumably, the results are substantially inferior to those obtained in the concussed patient sample because the MRI slice thickness in the non-concussed group was 3.75 mm. The intraclass correlation coefficient was found to be 0.51 for WM, 0.56 for GM, and 0.61 for CSF. In this lower-resolution sample, the segmentation method was estimated to be sensitive enough to detect percentage volume differences between MRI and CT which amounted to an average of ~7.1% (CI: [3.9, 10.3]%) for WM, ~6.2% (CI: [3.5, 8.9]%) for GM, and ~5.4% (CI: [3.1, 7.7]%) for CSF. The *d*_*S*_ metric was found to be 7.1 ± 4.12 mm (WM), 6.7 ± 3.9 mm (GM), and 3.4 ± 1.6 mm (CSF). Although the means and standard deviations of these quantities differ from those observed in the concussion group, the relationships between quantities recapitulate the findings illustrated in Figure 3 to indicate that *d*_*H*_ and *d*_*S*_ are directly proportional. Overall, these results thus confirm the necessity of validating CT-based soft tissue segmentations using MRI of standard, research-grade thickness (e.g., 1 mm) rather than MRI with slices of relatively large thickness (e.g., 3.75 mm).

## Discussion

### Feasibility

The ability to segment soft brain tissues from CT is largely dependent upon image contrast-to-noise ratio (CNR). In CT, the CNR itself depends on tube settings, iterative reconstruction method, radiation dosage and other factors; at standard dosages, the average radio-densities of GM and WM have been reported as 38.7 ± 2.2 Hounsfield units (HU) and 31.8 ± 2.3 HU, respectively (Craddock et al., 2006), resulting in an average X-ray attenuation of ~5 HU. Bier et al. (2016) similarly report radio-densities of 40.2 ± 3.3 HU (GM) and 28.48 ± 3.6 HU (WM) in their CT images, with the GM-WM radio-intensity difference being significantly different (*p* < 0.0001). The GM-WM CNR is reported as ~3 (Rapalino et al., 2012; Bier et al., 2016), but image filtering techniques have been reported to enhance the CNR by a factor as large as ~10 (Diwakar and Kumar, 2014; Bier et al., 2016). This allows the CT GM-WM CNR to compare favorably with the GM-WM CNR obtained from *T*_1_-weighted MRI at 3 T, where a meta-analysis found that single-slice and multi-slice MR images yield CNRs of ~18 and ~9, respectively (Fushimi et al., 2007). Together, these findings suggest that the delineation of the GM/WM boundary from CT is feasible using available CT technology. Nevertheless, it should be reiterated that, when available and of enough quality, MRI is by far preferable to CT for the purpose of brain soft tissue segmentation.

### Applications

Despite very limited previous research on CT brain tissue segmentation, there are numerous potential applications for this technology, including (1) the detection of brain pathology, (2) the measurement of brain volumetrics to assist studies of aging in health and disease, and (3) the quantitation of neuroanatomy in settings where MRI is undesirably expensive, unavailable or inaccessible. For example, the US Centers for Disease Control (CDC) report that the number of CT scanners exceeds that of MRI scanners by a factor which ranges between ~1.5 (developed countries) and ~5 (developing countries) (CDC, 2010). Furthermore, CT is more affordable than MRI and additionally constitutes the method of choice in certain clinical settings where image acquisition time is of the essence, such as neurocritical care (Williamson et al., 2017). For the latter reason, the availability of CT segmentation tools could be beneficial for TBI studies.

In stroke, TBI and other conditions which frequently involve CT, segmentation of images acquired with this modality could also be used to analyze perfusion imaging to study blood flow in the brain and to distinguish between the cores and penumbrae of cerebral lesions. CT-based volumetrics could also be useful in quantitative studies of brain atrophy associated with healthy aging, TBI or neurodegenerative diseases. Specifically, because the rate of brain atrophy in health differs from that observed in many diseases of the central nervous system, brain volumetrics can be used in conjunction with other anatomic and functional measures to estimate mortality risk and other parameters which are of substantial interest to clinicians, biomedical scientists, demographers, and epidemiologists.

Given that MRI availability in developing countries is relatively limited, software for CT-based brain segmentation could substantially extend the scope of certain large-scale epidemiological studies being carried out there. One such study is the Tsimané Health & Life History Project now underway in a region of rural Bolivia where MRI is logistically unfeasible yet where CT is available (Kaplan et al., 2017). Furthermore, because some patients cannot undergo MRI scanning due to claustrophobia, pacemaker implantation or other contraindications, techniques such as ours could facilitate the enrollment of these individuals in imaging studies. Given how transformative the research field of brain MRI processing has been over the past 30 years, the potential applicability of CT-based segmentation is thus clear.

### Comparison to Other Methods

There are very few other methods to which our approach can be compared quantitatively. One study which reports metrics like ours is that of Manniesing et al. (2017), where averages and standard deviations for *C*_*SD*_, *d*_*H*_, and *d*_*C*_ are reported for CT-only segmentations of WM and GM. In all cases, our results compare very favorably to theirs; for example, Manniesing et al. report 〈*C*_*SD*_〉 = 0.79 ± 0.05 and 〈*d*_*H*_〉 = 0.74 ± 0.26 mm for WM, where 〈〉 denotes the mean. In all three cases, our Sorensen-Dice coefficients are greater and the two distances quoted are smaller than theirs, as desirable; this statement also applies to the comparison of GM segmentations. By contrast, as expected, MRI-based segmentations clearly remain preferable. For example, Iscan et al. (2015) report that, for FreeSurfer-segmented GM, 〈*r*_*IC*_〉 = 0.88 ± 0.15 in a dataset of repeated MRI measurements. Furthermore, whereas our approach can—at its best— yield GM volume measurements which are within an average of ~5.4% of their MRI-derived values, the latter typically fall within <1% of their true values, on average (Eggert et al., 2012). Similarly, a comparison of the MRI- and CT-derived surfaces in Figure 1 easily indicates that only MRI-based segmentation can capture fine local variations in cortical shape, such as those due to gyri and sulci. In conclusion, our method could clearly benefit from refinement and from technology improvements to improve CT image SNR and CNR.

## Limitations

For clarity, our study's limitations can be divided into two groups, i.e., extrinsic or intrinsic. Extrinsic limitations involve factors pertaining to the imaging data themselves and which affect the efficacy of the method independently of it; such factors include radiation dose, the number of scans available, and the presence of metal objects inside the head. Since there is a direct—albeit nonlinear—relationship between radiation dose and SNR (Yu et al., 2009), we expect our algorithm to perform better if the data are acquired at higher radiation doses. Similarly, if repeated measurements are obtained, within-subject co-registration and averaging of CT volumes can improve SNR. If, on the other hand, metal objects (e.g., deep brain stimulation electrodes) are present inside the head, resulting artifacts may substantially compromise segmentation efficacy. One intrinsic limitation of our approach is the fact that, as Figure 1 illustrates, its ability to identify tissue boundaries correctly is suboptimal at brain locations where thin, long slabs of WM protrude into GM. Because the ability of our method to capture the geometric variability of the GM/WM interface is dependent upon GM/WM contrast, it results that the algorithm may not perform well in locations where the structure of the boundary is particularly complex. Improvements in the SNR and CNR between GM and WM can alleviate this drawback. A second limitation of this study involves the fact that *T*_2_-weighted MRI is preferable to *T*_1_-weighted MRI for quantifying water concentration in the brain. For this reason, the validation of CT-based CSF segmentations should be performed, if possible, based on the former MRI technique. Here, to circumvent this problem in the absence of *T*_2_-weighted MRI, we opted to compare ventricular CSF segmentations because brain ventricles are typically much larger than the CSF layer around the brain, especially in older adults. Nevertheless, future studies should strive to include *T*_2_-weighted MRI when undertaking validation of CT segmentations.

## Conclusion

The ability to segment soft brain tissues accurately from CT can substantially extend the utility of this important and cost-effective neuroimaging technique. Despite the limitations pertaining to the approach proposed here, our preliminary results indicate that reasonable segmentations of WM, GM and CSF can be obtained based on standard CT volumes of the human head. The methodological contributions described in this study can also be used as a foundation for the development of additional, more complex segmentation procedures for tasks such as the automated labeling of brain lobes and/or the identification of smaller structures such as gyri and sulci. Such refinements of our method, if feasible, would likely increase the utility of CT segmentation for brain imaging studies. Nevertheless, the accurate labeling of GM within thin gyri and of CSF within narrow sulci based on CT is likely to remain quite limited without substantial progress on CT technology to allow major improvements of image quality. When undertaking population-based studies of brain volumetrics calculated from CT data, researchers should duly account for the uncertainty of these measurements, especially in their statistical analyses. Such uncertainties are recapitulated by the magnitude of the variance in our Dice coefficients and Hausdorff distances, and this suggests that our ability to further refine our segmentation approach is largely predicated on the availability of CT head volumes with improved CNRs between WM and GM. MRI+CT data acquired from larger human samples are also required to improve the statistical estimates of our CT-based volume measurement errors relative to MRI.

## Author Contributions

AI conceived, funded and designed the study, implemented the segmentation approach, acquired imaging data, interpreted study results, wrote and coordinated the production of the manuscript. AM, KR, and NC contributed to the design of the study, analyzed imaging data, tabulated results, and contributed to their interpretation. DH contributed to the design of the study, identified eligible study participants, facilitated access to their neuroimaging data, and participated in the interpretation of study results. EL contributed to the design of the study, provided access to clinical data from eligible participants and participated in the interpretation of study results. All contributed approved the final version of the manuscript.

### Conflict of Interest Statement

The authors declare that the research was conducted in the absence of any commercial or financial relationships that could be construed as a potential conflict of interest.
